# Tracking Nano- and Microplastics in Plants: Uptake Pathways, Tissue Distribution, and Analytical Strategies from Microscopy to Spectroscopy

**DOI:** 10.3390/ijms27157019

**Published:** 2026-08-05

**Authors:** Abdullah Maqsood, Ewa Łobos-Moysa, Amna Jameel, Ewa Dacewicz

**Affiliations:** 1 Department of Water and Wastewater Engineering, Faculty of Energy and Environmental Engineering, Silesian University of Technology, Akademicka 2A Str., 44-100 Gliwice, Poland; ewa.lobos-moysa@polsl.pl; 2 Institute of Soil and Environmental Sciences, University of Agriculture, Faisalabad 38000, Pakistan; amnajameel4099@gmail.com; 3 Department of Sanitary Engineering and Water Management, Faculty of Environmental Engineering and Land Surveying, University of Agriculture in Kraków, Adam Mickiewicz Ave. 24/28, 30-059 Kraków, Poland; ewa.wasik@urk.edu.pl

**Keywords:** nano- and microplastics, phytotoxicity, plant–plastic interactions, microscopy, imaging techniques

## Abstract

Nano- and microplastics (NMPs) are now widely detected across agroecosystems and can act as physiological stressors in plants. Exposure occurs through contaminated soil, irrigation water, or airborne deposition, bringing particles into direct contact with roots and above-ground tissues. Reported entry routes include apoplastic transport, cracks formed at lateral root emergence, leaf stomata, and endocytosis once particles have crossed the cell wall. Once internalized, particles may translocate through the xylem and, in some cases, the phloem, accumulating in roots, stems, and leaves depending on particle size, surface charge, and plant structural characteristics. NMPs have been associated with oxidative stress, disrupted photosynthesis, and altered metabolic pathways. Detecting NMPs within heterogeneous, hydrated plant tissues remains challenging, as particles often show low contrast against biological structures and can be mistaken for cellular components. This review examines how microscopy techniques reveal NMPs size, surface attachment, tissue distribution, and cellular-level interactions, while noting that these approaches primarily provide morphological or localization information rather than confirming polymer identity. Complementary spectroscopic and mass-based analytical methods are discussed for their role in chemical confirmation and quantification. This review supports informed selection among imaging, spectroscopic, and quantitative techniques for studying plant–plastic interactions, while highlighting current analytical challenges facing the field.

## 1. Introduction

Plastic waste is widely considered a major threat to the environment [1,2]. It arises from the large-scale production of synthetic polymer materials and their widespread use, coupled with poor disposal practices [3,4,5,6]. Larger plastic items can fragment into smaller particles known as microplastics (MPs) and nanoplastics (NPs) through physical, chemical, or biological processes. These fragments are collectively called nano- and microplastics (NMPs). Their small size and resistance to breakdown allow them to spread widely in different environments [4,6,7,8]. According to recent data, NMPs have been reported to be present in large quantities in terrestrial systems, particularly in agricultural soil [5,9]. Plastics can enter the soil through various human activities. These include the application of sewage sludge, plastic mulch, wastewater irrigation, atmospheric deposition, and agricultural or consumer plastic degradation [10,11]. Therefore, plants that grow in contaminated soils may take up and translocate NMPs within their tissues [6,9].

The size of NMPs is small enough to interact directly with plant tissues, which is a growing concern regarding their uptake and movement in plants [9,12,13]. Experimental studies show that NMPs attach readily to plant surfaces, and, under the right conditions, can go on to enter through either the root system or aerial tissues. From there, particles can move through the vascular system and accumulate in roots, stems, and leaves; a handful of studies have also reported them in reproductive tissue, which matters given the obvious implications for food safety [7,13,14,15]. This presence may impact plant physiology and biochemistry. For example, NMPs can potentially alter the growth, induce oxidative stress, and disrupt metabolism and hormonal activities [3,7,13,16]. Although our understanding is increasing regarding plant–plastic interactions, it is still technically difficult to detect and characterize NMPs within plant tissues [10,11]. The tissues are structurally complex, highly hydrated, and chemically heterogeneous, which makes visualization challenging [17,18]. The particles vary from micrometres (µm) to nanometres (nm), and each size may require a different analytical approach [8,19]. Therefore, microscopic imaging techniques are considered important tools to study their uptake, location, and interaction with plant tissues.

Electron microscopy techniques allow visualization of the interaction of plastic with plants at various resolutions [8,19,20]. They can reveal features such as particles attached to roots and leaves, as well as their spatial localization within tissues and cells [21,22,23]. High-resolution structural details are obtained using scanning electron microscopy (SEM), field emission-scanning electron microscopy (FE-SEM), and transmission electron microscopy (TEM). These techniques are commonly applied to observe surface morphology, tissue interfaces, and ultrastructural changes potentially associated with NMP exposure [8,22,23,24,25]. Confocal laser scanning microscopy (CLSM) is used to visualize particles that are fluorescently labelled in tissues and can enable three-dimensional (3D) imaging of their localization [8,23]. Each instrument has its own strengths and limitations related to resolution, sample preparation, detection efficiency, and data interpretation [22,26,27]. However, none of these microscopy techniques can independently confirm polymer identity or quantify particle accumulation, which is why they are often combined with spectroscopic and mass-based analytical methods capable of chemical confirmation and quantification. This is why researchers often face challenges when selecting the most suitable imaging tool and complementary analytical strategy to examine the complex interactions between plants and plastics [28,29].

The number of studies on NMPs in plants are increasing, but our understanding remains limited regarding how these particles interact with plant cells. It is also challenging to determine which imaging, and analytical techniques can detect and characterize them most accurately under different experimental conditions. Here we bring together what is currently known about NMPs uptake and translocation in plants and ask how well the available imaging and analytical tools actually capture these processes. It also assesses the suitability of SEM, FE-SEM, TEM, and CLSM for visualizing these particles in plant tissues, alongside complementary spectroscopic (FTIR, Raman, AFM-IR, synchrotron FTIR, hyperspectral imaging) and mass-based (ICP-MS, Py-GC/MS) methods for chemical identification and quantification. These techniques imaging approaches are compared in terms of resolution, detection efficiency, sample preparation, chemical identification capability, and quantitative capability. We also highlight the major methodological limits and outline future research directions in plant–plastic studies.

## 2. Review Methodology

**Figure 1:**
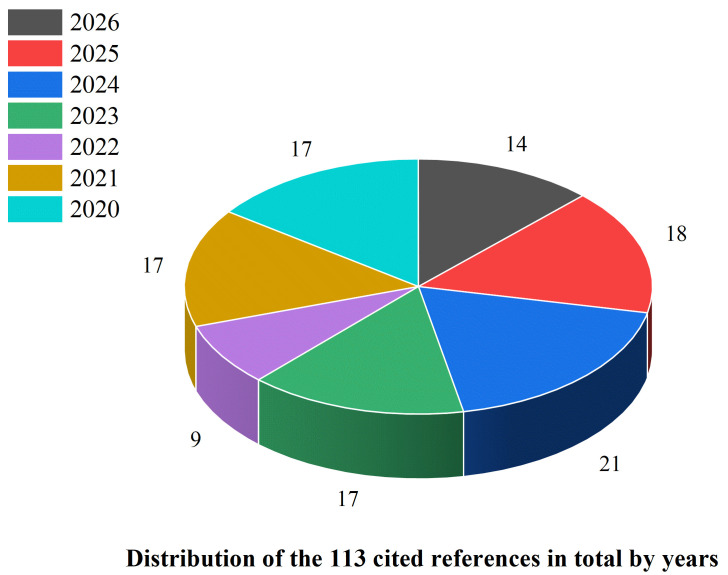
Distribution of the 113 cited references included in this review by year of publication (2020–2026).

We have searched literature by using PubMed, Web of Science, and Google Scholar, with keywords like “nano- and microplastics”, “phytotoxicity”, “microplastic”, “microscopy”, “plant–plastic interactions”, “nanoplastic”, “scanning electron microscopy imaging”, “analytical methods”, “field emission-scanning electron microscopy imaging”, “ultrastructural analysis”, “transmission electron microscopy imaging”, “instrumental analysis”, “confocal laser scanning microscopy imaging”, “FTIR spectroscopy”, “Raman spectroscopy”, “AFM-IR”, “synchrotron radiation-FTIR microspectroscopy”, “hyperspectral imaging”, “mid-infrared-hyperspectral imaging”, “chemical imaging”, “inductively coupled plasma–mass spectrometry”, and “pyrolysis gas chromatography/mass spectrometry.” Search terms were combined using Boolean operators, following representative strings such as (“nanoplastic” OR “microplastic”) AND (“plant” OR “root” OR “leaf”) AND (“microscopy” OR “FTIR” OR “Raman” OR “ICP-MS” OR “Py-GC/MS”). In the first search, 612 articles were found, and duplicate records across databases were removed prior to screening using reference management software Mendeley Desktop (version 1.19.8; Mendeley Ltd., London, UK). Then we manually reviewed every title, abstract, and full text to filter the results. The inclusion criteria were that the research study must be published in English, peer-reviewed, and focused on plant systems and microscopy imaging. Crucially, these papers reported how NMPs interact with plants, such as uptake, internal translocation, tissue localization, and phytotoxic effects. Another requirement was that the studies included microscopic, spectroscopic, or ultrastructural characterization using advanced imaging and analytical techniques. Following this screening process, 113 articles were selected for final inclusion, including original research articles, review papers, method articles, and protocol articles. The cited studies were published between 2020 and 2026 (Figure 1).

## 3. Plant Interactions with Nano- and Microplastics

Interactions between plants and NMPs are generally considered to occur at several stages. These may include entry into plant tissues, movement through vascular and cellular pathways, and physiological and biochemical responses in the plants. These mechanisms depend on particle characteristics and plant structural properties, which may collectively affect the level of uptake, internal distribution, and can contribute to stress under certain conditions. Detailed mechanisms are described in the following subsections.

### 3.1. Mechanisms of Uptake by Plant Systems

**Figure 2:**
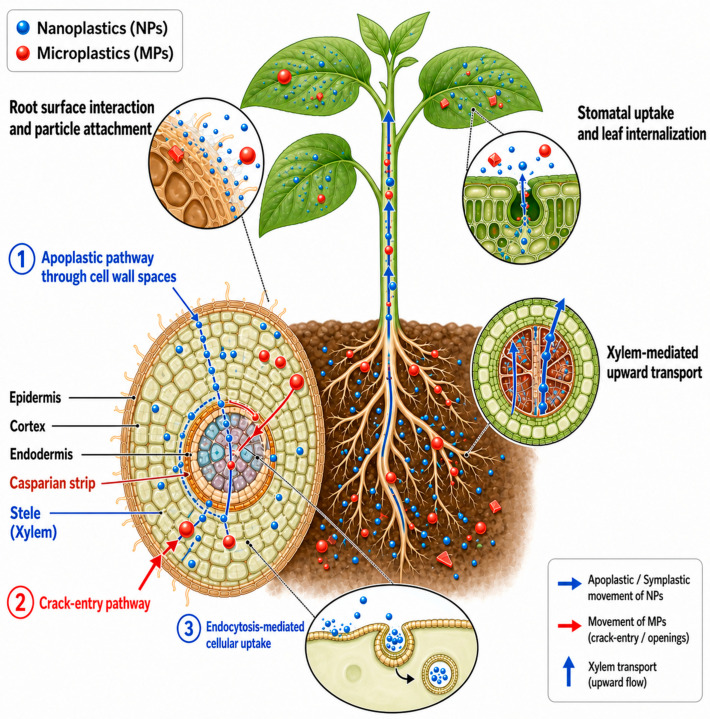
Mechanisms of nano- and microplastics (NMPs) uptake and transport in plant root and leaf systems. Particles interact with the root surface before entering via three routes: (**1**) apoplastic movement through cell wall spaces, restricted at the endodermis and Casparian strip; (**2**) crack-entry, permitting larger particles through natural root openings; and (**3**) endocytosis, enabling active cellular uptake of nanoplastics. Particles reaching the stele may be transported upward through the xylem via the transpiration stream, while atmospheric particles may enter leaves through open stomata. NPs, nanoplastics; MPs, microplastics. Figure created using the online scientific illustration platform Illustrae (https://illustrae.co/, accessed on 22 June 2026).

Plants appear to uptake NMPs mainly through their root system [30] (Figure 2). The root surface is covered with mucilage and exudates that can interact with plastic particles through physicochemical processes such as adhesion, aggregation, and electrostatic interactions, potentially leading to partial retention of particles at the root surface. This interfacial layer may act as a physical and chemical barrier that could influence particle attachment and entry into the root system, with effects likely depending on particle size, plant species, and root physiological conditions [22,31,32,33]. However, some particles have been reported to pass through this layer and reach deeper root tissues [6,30]. Sahai et al. [30] conducted a lab experiment on garden cress (*Lepidium sativum*) exposed to 100 nm polystyrene (PS) NPs across a range from 10 μg L^−1^ to 100 mg L^−1^ and found particle concentration within the root tissues ranging from 0.023 to 3.936 NPs μm^−3^. Particles small enough to pass the cell wall’s size-exclusion limit may then enter the cell via energy-dependent endocytic routes such as clathrin-mediated endocytosis and macropinocytosis [34]. Overall, root uptake of NMPs appears to be governed by structural and physiological properties of root tissues that influence particle attachment, entry, and internal transport [22,34].

NMPs can move through roots via the apoplastic pathway which follows cell wall spaces [8,9,20]. This route allows NPs and very small MPs to pass from the epidermis to within root tissues, particularly at lateral root emergence zones and other regions where apoplastic barriers such as the Casparian strip may be less developed. After reaching the xylem these particles can be transported upward by transpiration flow [30,35]. Wheat (*Triticum aestivum*) and lettuce (*Lactuca sativa*) grown hydroponically and exposed to PS particles (0.2–10 µm; 50 mg L^−1^) showed size-dependent uptake and transport. The 0.2 µm particles entered root vascular tissues (xylem) and were translocated to shoots, while 2.0 µm particles mainly entered at lateral root emergence sites via crack-entry zones. In that study, particles ≥ 5 µm showed minimal uptake. Translocation increased under higher transpiration rates, indicating transpiration-driven transport [35]. The NMPs may enter into roots mainly via physical cracks in root tissues [30,35]. Such openings may allow larger particles to bypass cell wall size limitations and reach internal tissues including the vascular system [15,22,30].

Particle size significantly affects uptake, as nanoscale particles can enter cells through endocytosis or move along apoplastic pathways, whereas larger MPs mostly remain on the root surface or enter only through cracks [6,9,36]. Researchers reported that in edible herb *Lepidum sativum*, 100 nm NPs were successfully taken up by the roots but their movement to the rest of the plant is highly restricted. Only 18% of the particles reached the stems and 13% reached the leaves, relative to the accumulation in the roots, serving as a reminder of how limited root-to-shoot movement often is [30]. Surface charge also appears to influence particle transport because negatively charged particles may move more easily through the apoplast, whereas positively charged particles tend to adhere to mucilage, cell walls, and root hairs [37,38]. For example, researchers found that in *Arabidopsis thaliana*, charged NPs show different accumulation patterns: positively charged particles mostly remain at the root surface, while negatively charged particles are more frequently observed in internal tissues such as the apoplast and xylem. Both types inhibit plant growth, with above-ground fresh weight reduced by 41.7% at 0.3 g kg^−1^ and 51.5% at 1.0 g kg^−1^ under negatively charged NPs exposure after seven weeks [37].

In addition to soil-based pathways, plants may be capable of absorbing NMPs through their leaves [5]. Small plastic particles may enter or accumulate around stomata, and in some cases may move into leaf tissues [20,39]. Nanoscale plastics may also pass through or between the layers of the cuticle. Leaf uptake is usually lower than root uptake, but it can become important when many particles are present in the air or irrigation water [3,21]. Airborne plastics can deposit on leaves and may enter through open stomata during gas exchange [6,40]. In a lab experiment, researchers exposed maize (*Zea mays*) leaves to demonstrate that stomata can allow atmospheric plastics to enter directly. When leaves received dust with 1.05 × 10^3^ µg/g of polyethylene terephthalate (PET), internal levels reached 4.47 × 10^3^ ± 0.53 × 10^3^ ng/g on day 5 and 7.76 × 10^3^ ± 2.28 × 10^3^ ng/g on day 10. Reducing the dust to 586 µg/g lowered accumulation to 502 ± 110 ng/g and 811 ± 240 ng/g after five and 10 days. This suggests that stomatal uptake may depend, at least in part, on dust concentration, with higher concentrations leading to more plastic inside maize leaves [40]. NPs may potentially travel through spaces between cells or attach to cell structures based on their size and surface characteristics [9,14,41]. Overall, particle size and surface charge consistently emerge as the main determinants of root uptake across these studies, though the available evidence spans a wide range of exposure conditions, from environmentally realistic to substantially elevated concentrations, and is derived almost entirely from controlled hydroponic or artificial dust-exposure systems rather than field soil conditions.

### 3.2. Internal Translocation and Tissue Accumulation

**Figure 3:**
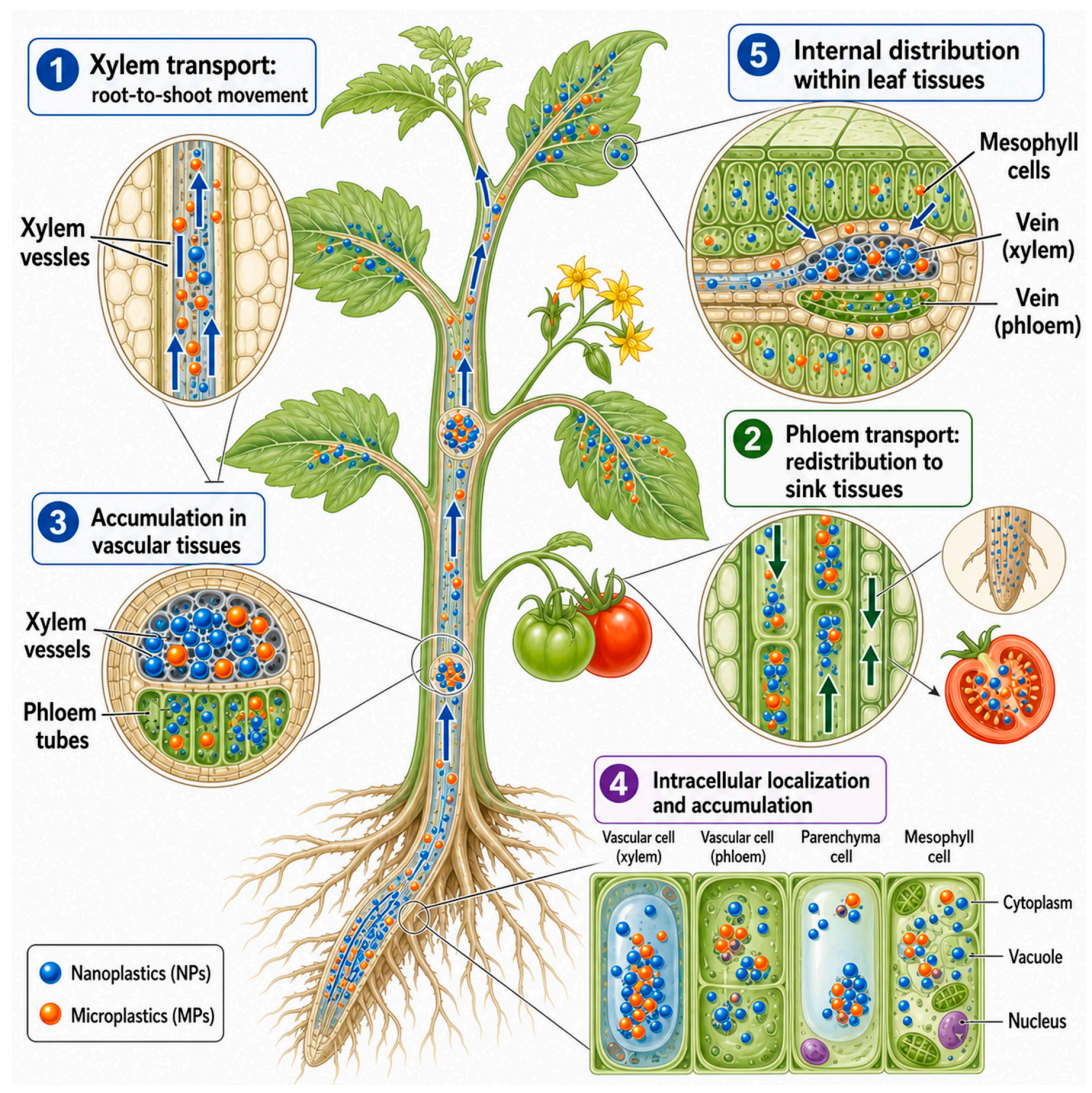
Internal translocation and tissue accumulation of nano- and microplastics (NMPs) in plants. (**1**) Xylem transport carries particles from root to shoot with the transpiration stream. (**2**) Phloem transport redistributes particles toward sink tissues, including root tips and developing fruit. (**3**) Particles accumulate within xylem vessels and phloem tubes of vascular bundles. (**4**) Intracellular localization occurs within the cytoplasm, vacuole, and other organelles of vascular, parenchyma, and mesophyll cells. (**5**) Within leaf tissue, particles are further distributed between xylem and phloem veins and surrounding mesophyll cells. NPs, nanoplastics; MPs, microplastics. Figure created using the online scientific illustration platform Illustrae (https://illustrae.co/, accessed on 22 June 2026).

Once NMPs enter plant tissues, they may potentially move to different organs through the vascular system [41] (Figure 3). In the xylem, particles are transported from roots to stems and leaves, and higher transpiration rates are generally associated with increase in their upward movement [20,35]. NMPs can also potentially travel through the phloem, which may enable movement in directions toward non-photosynthetic tissues, including roots and developing fruits [20,39]. In one hydroponic study, tomato (*Solanum lycopersicum*) plants received a foliar application of 70 nm PS-NPs, and after 7 days only 3.51% of the dose was recovered in the roots, suggesting that phloem transport to non-photosynthetic tissue is a fairly minor route [39]. Particles may accumulate in vascular bundles, stems, and other internal structures of tissues depending on their size, surface properties, and plant physiology [3,6].

Aerial plant tissues can potentially take up NMPs when particles accumulate on leaves. Some particles may attach to trichomes, stomata, or cuticular folds [20,42]. Depending on their surface charge, certain particles may remain on the surface, while others can potentially move into the epidermis or mesophyll [3,6]. For example, scientists reported that when tomato leaves were exposed to PS-NPs through foliar application (0.45–4.5 μg d^−1^ per leaf), positively charged particles appeared to enter the mesophyll more easily and showed a relatively uniform distribution within leaf cells [39]. After entering the tissue, NMPs may potentially reach the leaf vasculature and then move through the xylem or phloem. This pathway may enhance or contribute to their transport from roots to shoots [9,20]. In a hydroponic study, carrots (*Daucus carota*) were used and small PS particles (<0.2 µm) were found in leaves. Larger particles (0.607 and 1.219 µm) reached the leaves only when plants were co-exposed to 4 mg L^−1^ arsenic, which suggests that transport was strongly dependent on particle size and likely influenced by environmental conditions [43]. NMPs have been observed in the cytoplasm and vacuoles of plant cells and, in some cases, in other organelles. Instead of looking like single particles, they are mostly found in clusters, which may indicate that vesicle-based processes are involved in their intracellular movement [15,34,44]. The accumulation normally takes place in the areas which are involved in active vascular transport, such as root tips, stele tissues, leaf veins, and stem vascular bundles [5,30]. Diversity in particles movement is likely caused by the characteristics of the plants and the surrounding conditions [45]. Together, these findings point to xylem-mediated transport as the dominant translocation route, while the substantial variation in reported translocation efficiency, and its apparent sensitivity to co-contaminants such as arsenic, suggest that translocation potential is not governed by particle properties alone.

### 3.3. Physiological and Biochemical Responses to NMPs Exposure

**Figure 4:**
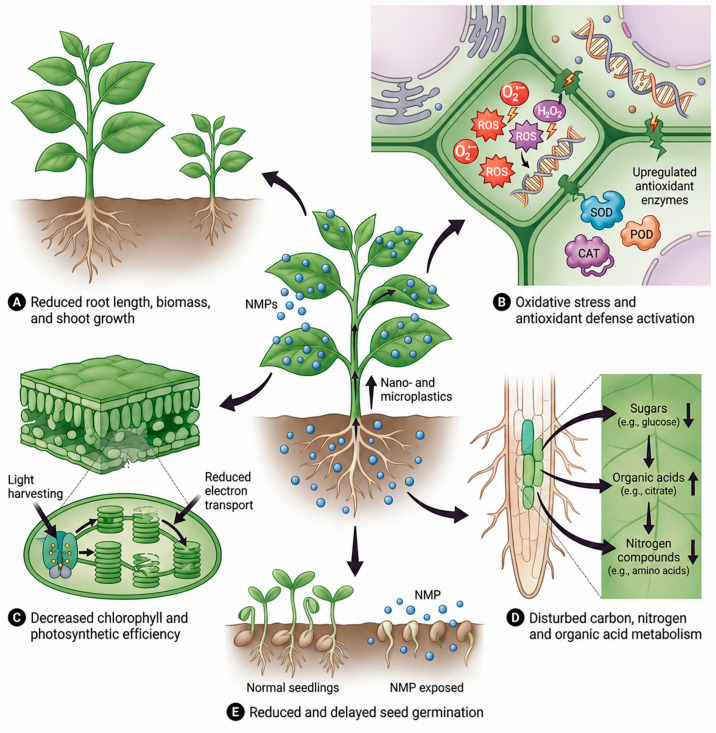
Physiological and morphological effects of nano- and microplastics (NMPs) on plants. (**A**) Reduced root length, biomass, and shoot growth following NMP exposure. (**B**) Oxidative stress response, showing accumulation of reactive oxygen species (ROS), including superoxide anion (O_2_•^−^) and hydrogen peroxide (H_2_O_2_), alongside upregulation of antioxidant enzymes superoxide dismutase (SOD), peroxidase (POD), and catalase (CAT). (**C**) Decreased chlorophyll content and photosynthetic efficiency, associated with reduced light harvesting and impaired electron transport within the chloroplast. (**D**) Disturbance of root carbon, nitrogen, and organic acid metabolism, including altered levels of sugars, organic acids, and nitrogen compounds. (**E**) Reduced and delayed seed germination in NMP-exposed seedlings compared with normal seedlings. Figure created using the online scientific illustration platform Illustrae (https://illustrae.co/, accessed on 22 June 2026).

The contact of plants with NMPs can lead to a number of physiological and biochemical changes [37,42]. Many studies have reported a decrease in growth such as root length, biomass, and shoot development [16,46,47] (Figure 4A). For example, in a laboratory experiment, foliar application of PS-NPs (0.1–1 mg L^−1^) on lettuce resulted in clear growth inhibition in plants. Comparing these plants to the control group showed a significant decrease in percentage of dry biomass (14.3–27.3%), plant height (24.2–27.3%), and leaf area (12.7–19.2%) [42]. We see comparable results in another study. When the seedlings of rice (*Oryza sativa L*.) were exposed to PS-NPs at 100 mg L^−1^, their primary roots only reached 7.8 ± 0.4 cm, whereas control plants reached 10.0 ± 0.7 cm. This suggests that PS-NPs were stressful and reduced root growth [14]. Mainly, these effects are likely connected with the changes in hormone regulation, especially in auxin and cytokinin pathways that control the root structure and shoot growth [14,48].

When plants uptake NMPs, they may experience oxidative stress at the biochemical level [46]. This happens because increasing reactive oxygen species (ROS) levels can affect cellular function by damaging nucleic acids, membrane lipids, and proteins [49]. In order to minimize this damage, plants often activate their antioxidant defence mechanism, primarily by increasing the activity of superoxide dismutase (SOD), peroxidase (POD) and catalase (CAT) (Figure 4B). The activation of these enzymes is an indicator of a putative protective response commonly observed in a stressed environment [47,50]. For example, seeds of onion (*Allium cepa*) have been treated by researchers with 50 nm PS-NPs for 72 h. The highest dose (1 g L^−1^) was reported to induce significant oxidative stress. Specifically, hydrogen peroxide (H_2_O_2_) levels reached 1.03 ± 0.31 mmol g^−1^ and lipid peroxidation reached 30.12 ± 1.99 nmol g^−1^ [15]. Another experiment reported that dandelion (*Taraxacum mongolicum*) seedlings grown in soil contained 1% w/w polyethylene (PE) MPs (200 μm). After 50 days, the seedlings showed higher oxidative stress, with superoxide anion (O_2_•^−^) increased by 41%, hydrogen peroxide by 44%, and malondialdehyde by 57%. Activities of SOD, POD, and CAT also increased by 44%, 49%, and 132%, respectively, which indicated a strong enzymatic response [47].

Photosynthetic processes are also generally considered to be sensitive to NMPs. Reductions in chlorophyll a, chlorophyll b, and carotenoids are commonly associated with decreased photosynthetic efficiency (Figure 4C). Additionally, shifts in the chlorophyll a/b ratio suggest changes in photosynthetic pigment composition, potentially reflecting adjustments in light-harvesting capacity [5,51,52]. Reduced carbon assimilation may also disturb electron transport in photosystem II and may reduce overall energy production [51,52]. Together, these effects are likely to contribute to decreased plant productivity. Teng et al. [52] conducted a soil experiment where tobacco (*Nicotiana tabacum* L.) seedlings were exposed to PE-MPs (0–1000 g·kg^−1^) for 48 days. Exposure significantly decreased chlorophyll content (4.3–14.0%), reduced ribulose-1,5-bisphosphate carboxylase/oxygenase (Rubisco) activity (4.23–30.9%), and lowered maximum photosynthetic rate (Amax) by 20–39.3%, indicating impaired carbon assimilation, electron transport, and light-harvesting capacity. Exposure to NMPs has been reported to affect plant metabolism in several ways. Altered levels of sugars, organic acids, nitrogen and carbon suggest that metabolic pathways may shift under stress (Figure 4D). Reduced carbon fixation can alter root metabolism and nutrient allocation, showing that the plant responds broadly rather than only in one part [46,47,51]. In an experiment, dandelions exposed to MPs showed strong metabolic changes. Sucrose and D-xylose increased under PE and polylactic acid (PLA) MPs, but D-xylose decreased under poly(butylene adipate-co-terephthalate) (PBAT) MPs. Levels of malic, tartaric, 2-butenedioic, glyceric, and citric acids rose sharply, by 14–21, 15–28, 47–78, 140–346, and 299–825 times, respectively. In addition, 4-aminobutanoic acid and L-serine increased under PE and PLA MPs, while L-serine declined under PBAT MPs. These changes indicate disrupted carbohydrate, organic acid, and nitrogen metabolism under MP stress [47].

Seed germination appears to be highly sensitive to NMPs stress [5,53] (Figure 4E). If germination is delayed or inhibited, seedlings may establish poorly, and their later growth can consequently be reduced [16]. A study examined how leachates from polycarbonate (PC) MPs affect garden cress seed germination. In a substrate-free system, exposure to new PC leachate reduced germination by 20% at 1:10 dilution, 54% at 1:1 dilution, and 77% when undiluted after seven days. Aged PC leachate showed a weaker effect, with inhibition between 6%, 13% and 18% depending on concentration. In a substrate system of seed growth, the inhibition was reduced to some extent. Under those conditions, new PC leachate caused 21–61% inhibition, while aged PC leachate resulted in only 3–19% inhibition [53]. Despite differences in plant species, polymer type, and exposure concentration, these studies consistently point to growth inhibition, oxidative stress, and reduced photosynthetic performance as shared physiological responses to NMP exposure, indicating that these effects may reflect a conserved stress pathway rather than species- or polymer-specific reactions.

## 4. Imaging Techniques for Detecting NMPs in Plants

**Figure 5:**
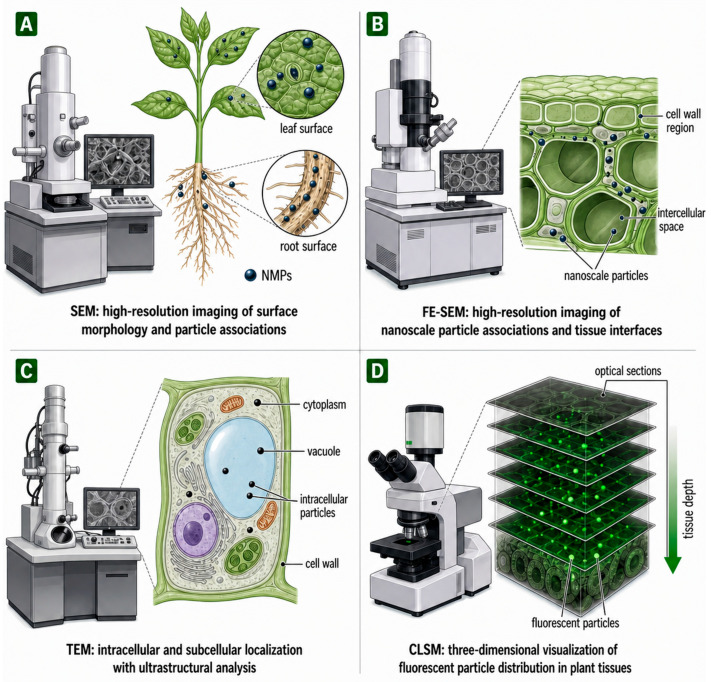
Overview of microscopy techniques used for detecting nano- and microplastics (NMPs) in plant tissues: (**A**) scanning electron microscopy (SEM), (**B**) field emission-scanning electron microscopy (FE-SEM), (**C**) transmission electron microscopy (TEM), and (**D**) confocal laser scanning microscopy (CLSM). These techniques enable visualization of particle tracking from plant surfaces to cellular, subcellular, and tissue levels. Figure created using the online scientific illustration platform Illustrae (https://illustrae.co/, accessed on 22 June 2026).

To understand the process of interaction between NMPs and plants, it is important to better characterize them and track their movement within tissues. Imaging techniques such as SEM, FE-SEM, TEM, and CLSM can be used to visualize specific aspects of these particle–plant interactions (Figure 5). Each technique differs in the signal it detects, the spatial scale and type of information it provides, and its ability to distinguish plastic from biological structures; sample preparation requirements also differ between techniques, and combining these approaches may provide a more complete view of how plastics affect plants. Table 1 provides an overview of selected studies that have used these microscopy techniques to detect and localize NMPs in terrestrial plants. A common limitation of the microscopy techniques discussed in this section is that they cannot independently confirm the polymer identity of an observed particle. SEM, FE-SEM, and TEM primarily provide morphological information, whereas CLSM detects fluorescence signals rather than the polymer itself. Therefore, microscopy observations should be interpreted together with complementary analytical techniques when confirmation of plastic identity is required.

### 4.1. Scanning Electron Microscopy (SEM) in Plant–Plastic Interaction

SEM is widely used to examine plant tissue morphology and to visualize interactions between plant tissues and NMPs [22,60,67]. It produces high-resolution images that show the shape, texture, aggregation, and spatial interaction of particles with plant tissues by scanning the sample surface with an electron beam [25,60,68]. SEM typically resolves features at the scale of a few to tens of nanometres [69], enough to reveal particle shape and surface attachment [3,20]. However, it does not confirm chemical or polymer identity on its own [70,71,72]. When plastic particles enter soil or the atmosphere, they may accumulate and cluster on roots, root hairs, leaf surfaces, stomata, and along epidermal cell boundaries. The imaging of these regions can be performed using SEM [3,20,38,44]. These images provide evidence supporting surface retention and attachment of particles; however, they do not provide definitive evidence of internal movement or translocation [3,7]. This capability is illustrated by a study, where SEM observations of cotton (*Gossypium hirsutum*) roots exposed to 300 mg L^−1^ PS-COOH for 24 h, performed using a Hitachi SU3500 SEM, showed the presence of MPs particles on the root surface [46]. Another hydroponic experiment exposed rice seedlings to 200 nm PS-MPs under concentrations of 0.1, 10, and 1000 mg L^−1^ for 14 days. SEM observations, performed at an accelerating voltage of 6 kV, a working distance of 8.0 mm, and magnifications of 200× and 500×, revealed the presence of MPs aggregates on the root epidermis and bud surfaces, primarily localized on external plant structures, indicating surface-associated retention of particles [50]. However, conventional SEM analysis alone cannot definitively exclude particle entry into root tissues or cells.

In a separate study, cucumber (*Cucumis sativus* L.) seedlings were exposed to PS-NPs of four particle sizes (100, 300, 500, and 700 nm). SEM imaging, performed using a Quanta-200 (FEI) instrument for initial observation followed by a higher-definition SU8000 (Hitachi) instrument for confirmation, revealed size-dependent behaviour: structures consistent with 100 nm particles adhered densely within root intercellular spaces with visibly altered morphology, while structures consistent with 500–700 nm particles retained their original shape and were also visualized in stems, leaves, calyx, and young fruit tissue; corresponding structures at 100 and 300 nm were not identifiable in flowers or fruits. The authors concluded that smaller particles undergo morphological changes during upward transport, likely due to mechanical shear stress or chemical interactions with plant substances, while larger particles remain structurally stable and accumulate further into aerial tissues [66]. SEM can also be applied to fractured plant tissues to examine internal tissue regions, such as cortical or vascular tissues, but it may not able to confirm whether particles have truly entered cells [22,67]. It provides high-resolution images of particle–surface interactions but has several inherent limitations [22]. Only a small area can be scanned at one time, which limits the acquisition of quantitative particle-count data [8]. The images are also in a grayscale and do not provide chemical or polymer-specific information [26,71]. Consequently, particles with similar morphology may be misidentified as plastic particles unless complementary analytical methods are used [26,69,71]. Overall, these studies illustrate that SEM reliably captures surface attachment and aggregation of NMPs across diverse plant species and exposure conditions, but its morphological nature means that findings of this kind should corroborate chemical identification techniques before particle identity is considered confirmed.

### 4.2. Field Emission-Scanning Electron Microscopy (FE-SEM) in Plant–Plastic Interaction

Compared with standard SEM, FE-SEM provides higher-resolution images and reveals finer surface details [24,73]. This makes it ideal for examining plant ultrastructure, specifically cell walls, membranes, and the nanoscale surfaces where NMPs interact [74]. FE-SEM improves on conventional SEM, resolving features at the scale of a few nanometres [75,76]. Like SEM though, it identifies particles by morphology alone and cannot confirm polymer identity [77]. Under appropriate sample preparation conditions, FE-SEM can visualize fine cell wall structures, including cellulose microfibrils and nanoscale surface features [78]. Observations of these structures can provide insights into the mechanisms by which particles attach to, persist on, and potentially accumulate within plant tissues [74,78]. For instance, in a study on green gram (*Vigna radiata*) and adzuki bean (*Vigna angularis*), FE-SEM, performed using a Zeiss Sigma-300 instrument, showed the presence of carbon-dot-embedded PS-NPs (100–223 nm) as aggregates on cell walls and within xylem vessels at both the lowest (6% v/v) and highest (100% v/v) concentrations tested, corroborating a concentration-dependent detection threshold established by complementary fluorescence microscopy. This suggests FE-SEM’s capacity to resolve nanoscale particle–tissue interactions may extend across a range of experimental exposure conditions [74].

In a laboratory experiment, researchers treated lettuce, carrot, and wheat with 100 nm fluorescent PS-NPs. FE-SEM analyses, performed using a JEOL JSM-7600f instrument at an accelerating voltage of 5 kV and selected to balance surface resolution against charging effects in the biological samples, showed particles associated with vascular bundles in lettuce roots, as well as clusters of particles within cortical cells in wheat roots, providing morphological evidence consistent with nanoparticle localization in internal tissues; however, FE-SEM imaging alone cannot definitively confirm intracellular uptake. The investigators examined at least four plants per species, with at least three tissue sections imaged per tissue type from a pool of 10 or more sections cut per plant, highlighting the capability of FE-SEM to visualize nanoparticles at high spatial resolution [23]. Beyond spatial localization, FE-SEM-based quantification has revealed that co-occurring contaminants can influence NPs accumulation in edible tissue: using a JEOL JSM7500 FE-SEM, PS NPs extracted from lettuce tissue via enzymatic digestion and membrane filtration were quantified by relating particle counts in the SEM images to an external calibration curve generated from tissue spiked with known PS-NPs concentrations, in hydroponically grown lettuce co-exposed to cadmium and 500 nm PS-NPs; shoot NPs concentrations were 67% higher than in plants exposed to NPs alone, suggesting that cadmium co-exposure enhances NP translocation from root to shoot. This finding highlights FE-SEM’s value not only for visualizing particle–tissue interactions, but also for quantifying how co-contaminants may alter NMP accumulation in food crops [79]. Although FE-SEM resolves finer surface and nanoscale features than conventional SEM, this resolution advantage does not extend to chemical specificity. Particles are observed by morphology alone, so polymer identity cannot be confirmed without complementary analytical methods [24,80]. Taken together, these findings show that FE-SEM is particularly valuable for resolving nanoscale particle–cell wall interactions that lie beyond the resolution of conventional SEM, but as with SEM, its morphological basis means that particle identity should not be assumed without independent chemical confirmation.

### 4.3. Transmission Electron Microscopy (TEM) in Plant–Plastic Interaction

TEM is an imaging approach that enables the observation of internal cellular and subcellular structures through the transmission of electrons across ultrathin samples [13,15]. TEM offers sub-nanometre resolution, the finest of the microscopy techniques covered here [81,82], but this comes with extensive sample preparation and a very small field of view [82,83]. As with SEM and FE-SEM, it cannot confirm chemical composition on its own [84]. When researchers study plants exposed to plastics, this technique is particularly valuable for investigating nanoscale interactions within cells. It allows the examination of changes in cell walls, membranes, vacuoles, mitochondria, and other organelles following exposure to NPs [13,15,42]. Importantly, TEM can reveal ultrastructural alterations associated with cellular stress responses induced, including vacuolization, organelle modifications, and membrane damage [13,85]. A laboratory experiment conducted on chicory seedlings (*Cichorium endivia* L.) exposed seedlings to PS particles of 20 nm and 200 nm. Analysis performed using a FEI Tecnai transmission electron microscope operated at an accelerating voltage of 100 kV, with particle dimensions measured using ImageJ software across at least 100 particles from a minimum of four different TEM images, showed that 65% of the 20 nm particles were smaller than 20 nm, with the most common size class (57%) ranging from 16 to 20 nm, whereas 91% of the 200 nm particles were below 200 nm, predominantly (50%) between 191 and 200 nm. Particle-like structures consistent with the 20 nm exposure material were observed in vacuoles and in the space between the plasmalemma and the cell wall, whereas structures consistent with the 200 nm particles were rarely observed. Particle morphology differed between treatments, with 20 nm particles appearing irregular and roundish, while 200 nm particles exhibited a more regular spherical shape. Ultrastructural changes, including plasmolysis, disruption of thylakoid membranes, and alterations in organelle morphology, were observed in treated cells [13]. Direct visualization of plastic particles by TEM can be challenging because most polymers exhibit weak electron contrast, and this limitation is further complicated by the extensive sample preparation required for ultrathin sectioning, which may introduce structural alterations or damage to the sample [26,81]. Heavy-metal staining improves ultrastructural contrast and may indirectly facilitate particle visualization; however, it can also introduce precipitation artefacts that visually resemble NPs, representing a significant source of false positives in nanoparticle identification within stained plant tissue [86].

TEM is not suitable for large-scale or highly quantitative analyses. However, it is particularly effective for providing evidence of intracellular localization and subcellular associations of NMPs [13,42,86]. For example, researchers investigated onion roots exposed to 50 nm PS-NPs at concentrations of 0.01, 0.1, and 1 g L^−1^. TEM analysis performed using a FEI Tecnai G2 Spirit electron microscope operated at an accelerating voltage of 100 kV, with particle dimensions measured using ImageJ software, showed an average major axis of 82.93 nm and a minor axis of 70.39 nm, with 77% of particles exhibiting both axes below 100 nm and 85% having at least one axis below 100 nm. TEM analysis of root tissue revealed electron-dense structures consistent with the exposure particles, ranging from 25 to 130 nm, often forming aggregates of two to five of such structures within vacuoles and the cytoplasm, with rare detection of similar structures as small as 25 nm within the nucleus. TEM further revealed ultrastructural alterations in treated cells, including an accumulation of electron-dense (likely lipid) bodies and impaired lipid mobilization, consistent with the internalization of PS-NPs in multiple cellular compartments [15]. In a study on carrot roots, TEM analysis showed that structures consistent with large-sized PS (>1 µm) rarely entered roots and were absent from leaves, whereas structures consistent with small-sized PS (50–150 nm) were more frequently visualized in both roots and leaves, localized mainly in intercellular spaces and pectin rather than inside cells. Under combined PS and As(III) exposure, TEM revealed a marked increase in the occurrence of particle-like structures consistent with PS, with such structures now found inside cells as well as intercellularly, and observed in leaves with diametres of ~600 nm [43]. This technique can also be used to characterize the size, morphology, and spatial distribution of individual particles associated with plant tissues, thereby supporting investigations of NMPs exposure and plant responses [15,86]. Collectively, these studies demonstrate that TEM provides the most direct evidence of intracellular and subcellular localization among the microscopy techniques discussed here, though its limited field of view and susceptibility to staining artefacts mean that individual observations are best treated as illustrative rather than representative of tissue-wide particle distribution.

### 4.4. Confocal Laser Scanning Microscopy (CLSM) in Plant–Plastic Interaction

The CLSM is a type of fluorescence imaging that is commonly used to examine the characteristics of cells and tissues in plants [8,30,86]. In NMPs studies, it is primarily used to detect fluorescently labelled particles and track their location within plant tissues [8,23,86]. CLSM resolution is limited by the diffraction limit of visible light, typically a few hundred nanometres [87,88,89], coarser than the electron microscopy techniques above [90]. Unlike those techniques, however, it enables 3D visualization of larger tissue volumes [91], but since it relies on fluorescent labelling, it confirms the presence of a label rather than the polymer itself [8]. These sections can be integrated to generate 3D images of internal structures [20]. It is a method that enables the precise localization of fluorescent signals associated with particles relative to plant structures such as the epidermis, cortex, stele, and vascular bundles [8,46]. For example, a 16-day hydroponic microcosm study in rice seedlings exposed to fluorescent PS-NPs used CLSM, performed with a Zeiss LSM880 confocal microscope equipped with Airyscan, using an argon laser for 488 nm excitation and a diode laser at 543 nm to distinguish PS-NP fluorescence from root autofluorescence, to visualize particle uptake and distribution within root tissues. Green fluorescent PS-NPs (~20 nm) were applied at concentrations of 10, 50, and 100 mg L^−1^, and CLSM imaging using 488 nm excitation revealed PS-NP-associated fluorescence signals within rice roots, indicating that NPs could enter and localize in root tissues. This study demonstrated the usefulness of CLSM for investigating the spatial distribution and internal localization of fluorescent NPs in plant tissues [14].

This method preserves tissue structure and maintains stable fluorescence signals, allowing researchers to rapidly examine particle distribution across relatively large tissue regions. CLSM differs from ultrastructural methods such as TEM and SEM, which generally require more complex sample preparation procedures [8,86]. This technique can help reveal spatial patterns of particle-associated fluorescence within specific tissue regions [30,92]. It may also assist in distinguishing signals originating from particles located within tissues from those associated with surface attachment, although this distinction often requires careful experimental controls and complementary evidence [86]. In a hydroponic foliar exposure study, CLSM imaging, performed using a Nikon AX confocal laser scanning microscope with a 561 nm excitation laser and emission collected within a 595–615 nm band, using 10× (NA 0.45) or 20× (NA 0.8) objectives with 1 μm Z-stack steps, revealed fluorescence signals associated with 100 nm fluorescent PS-NPs within specific tissues of lettuce, carrot, and wheat. The results suggested systemic translocation in lettuce, whereas particle-associated fluorescence in carrot and wheat appeared to remain largely restricted to root tissues [23].

Despite its advantages, CLSM has several limitations [86]. The imaging depth is limited in thickness and spatial resolution is restricted by the diffraction limit of light, making visualization of very small NPs challenging [24,86]. In addition, autofluorescence from plant materials such as chlorophyll, lignin, and phenolic compounds is a recognized source of background signal that can interfere with the detection of fluorescently labelled particles, and conventional dye-based labels remain susceptible to this interference. Free-dye leaching from labelled particles has been tested in several plant-uptake studies and found to be minimal under controlled conditions [8,18,93,94]. As a result, its observations are typically qualitative or semi-quantitative, and careful experimental controls are necessary to differentiate true particle-associated signals from background fluorescence [23,86]. In a laboratory foliar-exposure study, maize leaves were treated with fluorescent 30 nm PS-NPs for up to 10 days. CLSM, performed using a Zeiss LSM 880 confocal microscope with Airyscan, using dual excitation at 488 nm (plant tissue) and 532 nm (PS-NP fluorescence) with emission collected at 612 nm for the particle channel, revealed particle-associated fluorescence in the vascular tissue and near stomata, with control tissue showing no autofluorescence under the same conditions. Signal patterns across focal planes indicated the particles were located within the leaf rather than on its surface [40]. Taken together, these studies show that CLSM is well suited to tracking the spatial distribution and translocation of fluorescently labelled particles across intact tissue, but its reliance on labelling and susceptibility to autofluorescence mean that its findings are best interpreted alongside label-independent or chemically confirmatory techniques.

### 4.5. Quantitative Image Analysis and Particle Detection in Microscopy-Based NMP Studies

While microscopy techniques are mainly used to visualize NMPs localization and interactions with plant tissues, image analysis approaches can provide additional quantitative information. For SEM and FE-SEM images, segmentation methods such as threshold-based and manually assisted approaches are commonly applied to distinguish particles from surrounding structures, enabling estimation of parameters including particle number within analyzed fields, size distribution, area coverage, and morphological features such as circularity and aspect ratio [69,71,95]. TEM-based quantification generally focuses on individual particle characteristics, including particle dimensions and size distribution, due to its high resolution but limited imaging area [13,95,96]. In CLSM analysis, quantification is often based on fluorescence-derived measurements, such as fluorescence-positive areas or signal intensity, rather than direct particle counting [91]. However, intensity-calibrated approaches have been developed in specific cases to estimate particle numbers from fluorescence data [30].

However, quantitative microscopy analysis remains challenging due to variability in image quality, segmentation procedures, and particle recognition [69,71,95]. Automated detection may overestimate or underestimate particle numbers when particles overlap, form aggregates, or cannot be clearly distinguished from biological structures [71,86,95]. In addition, the limited field of view of high-resolution microscopy may affect the representativeness of quantitative measurements [8,19,86,96]. Therefore, transparent reporting of image-processing parameters and validation using complementary techniques are important for improving the reliability of microscopy-based quantitative assessments [19,23,71]. A comparative summary of the analytical characteristics of the microscopy techniques discussed above, alongside the complementary chemical identification and spatially resolved imaging methods described in Section 6, is provided in Table 2.

## 5. Sample Preparation and Technique-Specific Limitations in Plant Imaging

**Figure 6:**
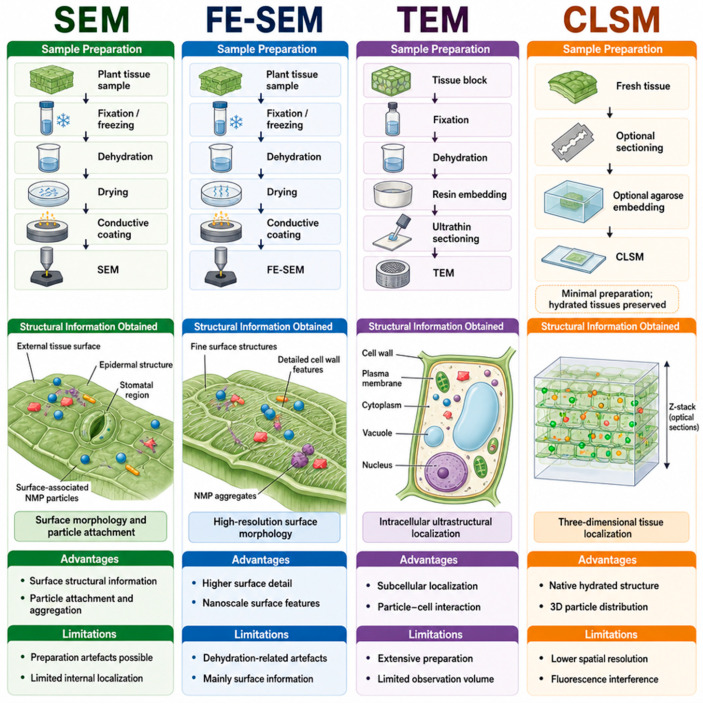
General workflow of plant sample preparation for microscopy-based detection, highlighting the main preparation steps, advantages, and limitations associated with scanning electron microscopy (SEM), field emission-scanning electron microscopy (FE-SEM), transmission electron microscopy (TEM), and confocal laser scanning microscopy (CLSM). Figure created using the online scientific illustration platform Illustrae (https://illustrae.co/, accessed on 22 June 2026).

Sample preparation plays a critical role in the reliable visualization of NMPs in plant tissues [86,97]. It affects how well tissue structure is preserved, the apparent localization of particles, and how images are interpreted [29,86]. High-resolution microscopy often requires tissues to be exposed to vacuum conditions or electron beams; therefore, preparation generally requires a balance between preservation of native structure and the technical requirements of the imaging method [86,98,99]. Internal tissue analysis commonly involves embedding samples in agarose or resin, followed by sectioning [29,86,100] (Figure 6). Although this approach helps preserve section thickness, it may displace loosely attached particles or obscure tissue–particle boundaries [86].

In conventional SEM, sample preparation typically involves fixation, dehydration, and conductive coating to preserve structural integrity and minimize preparation-induced artefacts, such as shrinkage or surface distortion [98,99]. For example, in a study on foliar-applied NPs in maize, leaf samples were excised, sectioned into small pieces, and frozen in liquid nitrogen, then freeze-dried and coated with gold for 60 s to improve conductivity. The cross-sections were examined using a SEM (Quanta 200, FEI) operated at an accelerating voltage of 20 kV in high-vacuum mode with backscatter detection, revealing NPs aggregates on and near the stomata of the leaf surface [7]. A representative SEM preparation procedure for cucumber leaves was described by Huang et al. [101]. Leaf tissues near the primary veins were collected and cut into small pieces at a laboratory in China. The samples were rapidly frozen in liquid nitrogen for approximately 6 h, then vacuum freeze-dried to preserve surface characteristics. Once dried, the samples were coated with a thin gold layer (approximately 1 nm) using sputter coating for 60 s to improve conductivity. FE-SEM has similar preparation requirements, including dehydration and conductive coating; however, these steps can produce artefacts, particularly in hydrated tissues [23,74]. For example, a study using fluorescent carbon-dot-embedded PS NPs in green gram and adzuki bean prepared root and stem sections by freeze-drying, followed by gold–palladium coating for 5 min, before FE-SEM (Sigma-300, Zeiss) observation. FE-SEM revealed PS NPs aggregates on the cortical cell walls and within xylem vessels, corroborating the localization pattern observed by fluorescence and confocal microscopy [74]. Another study on lettuce, carrot, and wheat, plant sections and whole mounts were fixed in 2.5% glutaraldehyde, dehydrated through a graded ethanol series, and critical-point dried. Samples were mounted on stubs and coated with a 10 nm platinum/palladium layer before imaging. FE-SEM revealed structures consistent with PS-NPs adhered to the inner surfaces of vascular bundles and aggregated within cortical cells of the roots [23].

Preparing samples for TEM is typically more labour-intensive and time-consuming [86,99]. This process includes fixation, dehydration, resin embedding, and ultrathin sectioning, which may introduce preparation artefacts and can limit the observable area [83,86,100]. Muccifora et al. [13] demonstrated this approach using endive (*Cichorium endivia* L.) roots and shoots. After cutting the tissues into small cubes, they pre-fixed them in Karnovsky solution to preserve tissue structure. The samples were subsequently post-fixed in osmium tetroxide, dehydrated through graded series, and embedded in an Epon 812-Araldite A/M resin mixture. Thin sections were then prepared and stained with uranyl acetate and lead citrate. In contrast, CLSM generally requires less extensive sample preparation. Fresh or lightly sectioned tissues can often be imaged directly without fixation, dehydration, or conductive coating [8,23,67,86]. In a laboratory study, Vleeming et al. [23] applied this approach to carrot, lettuce, and wheat seedlings. Root segments, basal stems, and leaves were collected, rinsed, and embedded in low-melting-point agarose. Sections of 60 μm thickness were prepared using a vibrating microtome (Leica VT100S) and mounted directly for imaging using a CLSM. In short, no single sample preparation protocol is universally applicable for NMPs imaging in plant tissues. Instead, each approach requires balanced structural preservation, particle retention, and analytical feasibility.

## 6. Integrated Approaches with Spectroscopic and Mass-Based Methods

The imaging techniques described above address complementary rather than overlapping questions, ranging from surface attachment and morphology to spatial translocation and intracellular localization, but none can independently confirm polymer identity or quantify particle accumulation and particle-like structures observed by microscopy and may be mistaken for plastics due to interference from biological material of similar size or morphology [19,20]. To overcome this, microscopy is commonly combined with spectroscopic and mass-based techniques, which serve two related purposes: confirming that the particles used in exposure experiments are chemically authentic before imaging begins, and, in some studies, directly detecting or quantifying particles recovered from plant tissue after exposure [26,49,102].

### 6.1. Chemical Identification: Point-Based, Hybrid, and Spatially Resolved Spectroscopy

Chemical confirmation of NMPs in plant systems is achieved through two complementary approaches: point-based and hybrid techniques that confirm polymer identity at a single location and spatially resolved techniques that map chemical signals across intact tissue. These are discussed in turn below.

#### 6.1.1. Point-Based and Hybrid Chemical Identification

Fourier-transform infrared (FTIR) spectroscopy and Raman spectroscopy are the most widely used tools for this chemical confirmation role [2,49,103]. In a study on cotton, FTIR spectroscopy was used to confirm the chemical identity of carboxyl-modified PS-MPs (PS-COOH) before root exposure experiments. FTIR spectra collected over the 400–4000 cm^−1^ range showed characteristic absorption bands at 698, 1450, 1490, and 1620 cm^−1^ associated with PS aromatic structures, along with peaks at 2930, 3020, and 3060 cm^−1^, and carboxyl-related bands at 1730 and 3460 cm^−1^, confirming the polymer composition of the particles subsequently used to demonstrate apoplastic uptake in cotton roots via confocal microscopy [46]. Similarly, in a study on rice, FTIR confirmed the identity of the PS-MPs used in exposure experiments, showing characteristic -CH_2_- absorption peaks at 717.450, 1471.544, and 2916.087 cm^−1^ that closely matched a standard PS reference spectrum; particle distribution on rice seed, root, and bud surfaces was separately showed by SEM [50]. In a study on wheat, Raman spectroscopy, performed using a Renishaw InVia Reflex Laser Confocal Raman Spectrometer over a 100–3500 cm^−1^ scanning range, the same verifying role prior to exposure, showed distinct peaks at 3045 cm^−1^ (aromatic C–H), 1587 cm^−1^ (asymmetric C–C stretching), and 987 cm^−1^ (ring breathing vibration) [58].

Beyond confirming test-material identity, Raman spectroscopy can also detect plastic particles directly within plant tissue. Later work on peanut and rice, using a Hooke Instruments HOOKE P300 Raman spectrometer with 785 nm excitation and a 5 mW laser power over a 200–3000 cm^−1^ range, detected 80 nm PS-NPs within the grains using the characteristic PS peak at 998 cm^−1^, providing direct chemical evidence that these particles had penetrated plant tissue [60]. Coupling microscopy with either Raman or FTIR therefore provides both physical and chemical information on the same particles, the shape and location from imaging, and the chemical identity from spectroscopy [104,105].

Hybrid methods extend this chemical confirmation to smaller spatial scales than either technique achieves alone. SEM–Raman and atomic force microscopy–infrared spectroscopy (AFM-IR) allow chemical analysis at very small spatial scales by merging high-resolution surface imaging with molecular identification, helping to distinguish plastics from biological or mineral material of similar size [70,102]. AFM-IR extends this further by measuring local infrared absorption while simultaneously examining surface structure at the nanoscale [40,106]. Using AFM–IR, performed with a Bruker IconIR instrument, researchers identified PET NP particles larger than 40 nm, and separately detected nano-sized PS particles, in digestive solutions prepared from field-collected leaves at heavily polluted sites (a Dacron factory for PET and a landfill site for PS), demonstrating that NMPs can be chemically confirmed after recovery from real leaf material, not only from laboratory-exposed model plants [40].

In summary, FTIR, Raman, and AFM-IR each confirm polymer identity reliably at a single point or particle, either before exposure experiments or after recovery from tissue, but none provide information on how a chemical signal is distributed across intact tissue.

#### 6.1.2. Spatially Resolved Chemical Imaging

Synchrotron radiation-FTIR (SR-FTIR) microspectroscopy extends conventional point-based FTIR to spatially resolved chemical imaging at the micrometre scale. In cherry radish (*Raphanus sativus*) root tissue exposed to 200 nm PE NPs, SR-FTIR imaging performed at the Shanghai Synchrotron Radiation Facility (BL01B1 beamline, Thermo Nicolet 6700 spectrometer, 1 µm step size, 4 cm^−1^ resolution) mapped functional group distribution across root cross-sections, showing aliphatic-C signal concentrated in the inner root tissue, carboxylic groups distributed throughout the root, and polysaccharide signal localized to the outer root margin. This demonstrates that SR-FTIR can resolve the spatial distribution of specific chemical functional groups within plant tissue at micrometre resolution, without requiring fluorescent labelling [107].

Visible/near-infrared-hyperspectral imaging (VNIR/NIR-HSI) has also been applied to characterize plant biochemical responses to NP exposure, rather than direct visualization of the particles themselves. In lettuce exposed to 20 and 200 mg kg^−1^ PE NPs in soil, hyperspectral imaging using a VIS-NIR camera (Middleton Spectral Vision) capturing 473 wavebands across 400–998 nm at 1.26 nm spectral resolution detected a significant reduction in the normalized difference vegetation index, alongside an increase in the plant senescence reflectance index, at the lower exposure concentration, consistent with reduced vegetative health and photosynthetic efficiency [108].

These two imaging-based spectroscopic approaches have also been combined and applied directly to microplastic-exposed crop tissue. In rice seedlings exposed to PET, PS, and polyvinyl chloride (PVC) MPs (0, 10, and 100 mg/L), VNIR-hyperspectral imaging (380–1013 nm, 2.2 nm spectral resolution) of leaves enabled detection of MPs stress levels with over 93.88% classification accuracy. Complementary SR-FTIR microspectroscopy, performed at the BL01B beamline of the National Synchrotron Radiation Laboratory (Bruker Vertex 70v spectrometer with Hyperion 3000 microscope, 4000–800 cm^−1^, 4 cm^−1^ resolution, 20 × 20 μm^2^ mapping areas), revealed PET-associated spectral features, including the ester C=O stretch (1730 cm^−1^) and benzene-ring bending vibration (841 cm^−1^), with substantially stronger signal intensity in leaf vein tissue than in mesophyll, indicating that PET preferentially accumulates within the vascular bundle rather than surrounding leaf cells [105]. Compared with the electron and fluorescence microscopy, which are largely limited to morphology or fluorescent-tag distribution [8,19,26], these spectroscopic imaging techniques provide direct chemical mapping of polymer-specific signals at the cost of coarser spatial resolution [105,107,108].

Mid-infrared-hyperspectral imaging (MIR-HSI) is an emerging extension of infrared chemical imaging, distinct from the point-scanning approach of conventional FTIR microscopy. It has been reported to combine wide-field acquisition with substantially reduced imaging time relative to conventional FTIR microscopy, while retaining polymer identification based on mid-infrared vibrational fingerprints in complex biological tissue. This allows spatial location and polymer identity to be obtained from the same acquisition [109]. It differs from VNIR/NIR-HSI, which reports biochemical and physiological signals through overtone and combination bands rather than direct fingerprint-region chemical identification [105,109]. To our knowledge, MIR-HSI has not yet been applied to plant tissue.

Building on this, SR-FTIR, VNIR/NIR-HSI, and MIR-HSI extend chemical confirmation across intact tissue by mapping chemical signals spatially rather than at a single point. MIR-HSI has demonstrated this combined capability in other biological matrices, but not yet in plant tissue. As with the point-based and hybrid techniques above, however, none of these spatially resolved approaches can determine how much plastic is actually present.

### 6.2. Quantitative Mass-Based Methods

Quantitative methods such as inductively coupled plasma–mass spectrometry (ICP-MS) and pyrolysis-gas chromatography/mass spectrometry (Py-GC/MS) address the remaining gap left by both microscopy and the spectroscopic techniques above: they estimate how much plastic is present, rather than only where it is or what it looks like [102,110,111]. Py-GC/MS identifies polymers from their thermal degradation products; for plant and other biological samples specifically, this generally requires more extensive pretreatment to remove organic interference than for cleaner sample types [2,20,112]. In basil (*Ocimum basilicum*), basil tissue was digested using hydrogen peroxide with an iron(II) sulfate catalyst prior to analysis, and Py-GC/MS analysis using an Agilent 8890 GC-MS coupled to a CDS Pyroprobe 6150 pyrolyzer (500 °C pyrolysis, HP-5MS column) identified PS via its styrene trimer fragment (m/z 91, 312), with limits of detection of 0.9, 0.3, and 0.7 μg g^−1^ and spiked recoveries of 84.8–96.0%, 64.8–102%, and 75.1–97.6% in roots, shoots, and leaves, respectively. Measured PS concentrations were 4.7 μg per 100 g in roots, 2.4 μg per 100 g in shoots, and 3.6 μg per 100 g in leaves, consistent with a concentration-gradient-driven root-to-shoot transport pattern [113].

ICP-MS is especially useful when plastics carry elemental labels, allowing direct, quantitative tracking of uptake and movement within plant tissue [2,68,102,112]. In wheat and lettuce grown hydroponically across a concentration series (5–5000 μg L^−1^) of europium-doped (PS-Eu) polystyrene NPs, ICP-MS showed that most particles accumulated in the roots, with root concentrations ranging from 1.3 to 1494 μg g^−1^ in wheat and 4.1 to 2220 μg g^−1^ in lettuce depending on exposure level; at the highest exposure concentration (5000 μg L^−1^), transfer to shoots remained below 3% of total uptake. Plants grown in soil (1–10 mg kg^−1^ PS-NPs for 14 days) showed much lower root concentrations (3.0–5.2 μg g^−1^ in wheat and 4.3–15.2 μg g^−1^ in lettuce), illustrating how growth medium affects measured accumulation [68]. These quantitative techniques support the spatial evidence obtained by microscopy, but cannot themselves describe particle location or shape within tissue [110,111,112], as summarized in Table 2. Taken together, ICP-MS and Py-GC/MS close the remaining gap left by both microscopy and spectroscopy, confirming that a full characterization of NMPs in plant tissue, covering location, chemical identity, and quantity, requires the combinination of several of these complementary techniques rather than relying on any single method. Table 3 summarizes these complementary analytical stages, from morphological observation through chemical identification, spatial chemical mapping, and quantification.

## 7. Conclusions and Future Perspectives

The NMPs are considered an emerging concern for agriculture and food safety. Rather than acting as simple physical barriers, crops have been shown to potentially uptake, transport, and accumulate these particles. This absorption may influence the physiology and metabolism of plants, and this could consequently affect their growth, yield, and nutrient utilization. These observations suggest that plants may play an active role in the environmental fate and transport of NMPs. Important details regarding the location of particles and interactions in plant tissues have been demonstrated by microscopy; however, each technique has limitations. Surface morphology is revealed by SEM, and more detailed images of surface features at the nanoscale are given by FE-SEM. For investigation of internal cell structures or localizing particles, TEM is commonly used as a standard approach, whereas CLSM helps in a complementary way by tracking translocation and tissue distribution in 3D. However, reliable polymer identification, complex sample preparation, and quantitative measurement remain challenging because microscopy techniques alone cannot independently confirm plastic identity. These limitations converge on three unresolved methodological challenges: distinguishing genuine particle internalization from surface attachment or artefactual deposition, obtaining chemical identity and spatial localization simultaneously rather than through separate techniques, and achieving reliable quantification within heterogeneous, hydrated plant tissue. To address this, it is necessary to combine high-resolution imaging with spectroscopy and mass-based analysis. Spectroscopic techniques such as FTIR and Raman are typically used to confirm the chemical identity of test particles, either before plant exposure or after recovery from plant tissue, while mass-based methods such as ICP-MS and Py-GC/MS provide quantitative estimates of particle accumulation that imaging alone cannot supply.

Future studies should be directed towards better understanding the entry of NMPs, their movement, and accumulation in plants, as well as their role in plant physiology and biochemistry under natural conditions. Comparisons between studies are currently difficult, largely due to differences in particle size, concentration, exposure conditions, labelling strategy, sample preparation, and criteria used to confirm particle localization across studies. This may be improved by adopting standardized methods for sample handling, imaging, and clear presentation of results, alongside consensus reporting guidelines and validation criteria for confirming true particle localization. Chemical and quantitative analysis of plastics in plant tissues can potentially be improved using new imaging methods, including spatially resolved, label-free chemical imaging approaches such as synchrotron radiation-FTIR microspectroscopy, which provides simultaneous spatial and chemical information. VNIR/NIR-HSI reports plant biochemical and physiological responses rather than direct polymer identity, whereas MIR-HSI reports direct polymer identity but has not yet been demonstrated in plant tissue. Cryogenic electron microscopy approaches may further reduce preparation-induced artefacts and better preserve native hydrated tissue structure. Integrating these approaches could help to better understand plant–plastic interactions and may improve assessment of potential risks to plants and food safety.

## Figures and Tables

**Table 1:** Sample preparation protocols and detection outcomes for microscopy-based visualization of nano- and microplastics (NMPs) in plant tissues.

Detection Technique	Terrestrial Plants (Part)	Sample Preparation	Type	Shape	Size (nm)	Research Result	References
SEM	Rye (root, stem, leaf)	Fixed in 2.5% glutaraldehyde (24 h) → dehydrated → critical-point dried → gold-palladium sputter-coated	PS	Spherical	100	Particle-like structures consistent with PS-NPs distributed within intercellular spaces and vascular bundles (xylem, phloem) of root, stem, and leaf, with lesser occurrence inside cells, structures showed partial deformation	[54]
SEM	Wheat (germinating seed)	Soaked in DI water or PET–fSPION suspension (24 h) → fixed in 4% formaldehyde (24 h) → rinsed with PBS → manually bisected → dried at 50 °C (6 h) → mounted with silver conductive adhesive → platinum-coated (~5 nm, 40 s, 40 mA)	PET–fSPION	—	—	Aggregates consistent with PET–fSPION localized in embryo (shoot apical meristem, radicle, coleoptile, plumule, scutellum), endosperm, and crease regions	[55]
SEM	Tomato (leaves, roots)	Fixed in 4% glutaraldehyde (48 h) → dehydrated (graded ethanol series, 30–100%) → mounted on slides → air-dried	PS-Eu (europium-doped PS)	—	—	Structures consistent with PS-Eu particles observed as aggregates on leaf surfaces, only a few such structures observed in roots (dispersed or in small clusters)	[39]
SEM	Chrysanthemum coronarium (root, stem, leaf)	Fresh tissue cut into small pieces → pre-fixed in 2.5% glutaraldehyde → washed (3×) with PBS → post-fixed in 1% osmium tetroxide → dehydrated (graded ethanol series, 30–100%) → critical-point dried → coated with gold-palladium (ion sputter, 50 s)	PS	—	—	Structures consistent with PS-MPs observed in root and stem intercellular spaces	[56]
SEM	Tomato (root)	Roots fixed in 4% glutaraldehyde (24 h, 4 °C) → dehydrated through graded acetone series (20–100%) → air-dried → mounted on stubs with double-sided carbon tape → gold-coated → imaged	PVC	—	<10	SEM revealed particle-like structures consistent with PVC-MPs inside root cells	[57]
SEM	Wheat (roots, leaves)	Fixation → dehydration → transverse sectioning → gold coating (~1 nm)	PS	Spherical	100	Structures consistent with PS-NPs observed dispersed in root and leaf vein tissues in transverse sections	[58]
SEM	Black gram (root)	Germinated seedlings sealed in 2 mL Eppendorf tubes with paraffin wax (pin-holes for moisture release) → frozen at −80 °C → freeze-dried (6 h, lyophilizer) → imaged	PE	Irregular	6–600	Particle-like structures observed adhered to the root surface of black gram	[59]
SEM	Peanut and rice (grains, roots, spikes)	Washed → frozen in liquid nitrogen → freeze-dried → gold sputtering	PS	Spherical	82.6	PS-NPs observed inside grains, roots, and spikes; localized in tissues and starch granules	[60]
SEM	Brassica rapa (leaf, stomata)	Freeze-dried → leaf slices affixed to conductive tape → gold-coated (sputter coater, 60 s, repeated 3×)	PVC	—	—	Particles observed within and on the outer surface of leaf stomata at day 5, blocking and stretching stomata; fewer particles present within stomata by day 15, with stomata appearing wizened; particles rarely observed within stomata by day 30, with stomata broken and closed	[61]
FE-SEM	Sweet pepper (roots)	Roots washed with ultrapure water → fixed in 10% formalin → embedded in paraffin wax → sectioned at 4 μm thickness	PS	—	—	Cross-sectional FE-SEM showed accumulation of structures consistent with MPs within root tissue in both 50 mg L^−1^ exposure groups; individual particle diametres within tissue were ~0.45–0.55 μm (0.5 μm MPs group) and ~0.8–1.2 μm (2.0 μm MPs group), indicating a critical size threshold of ~1.2 μm for root uptake	[62]
FE-SEM	Soybean (root)	Harvested → rinsed 5× with deionized water → sonicated 5 min	PLA	—	—	Particles adsorbed on root surface at day 2; by day 15, particles observed penetrating root cell walls and integrated with root cells	[63]
FE-SEM	Lettuce (roots)	—	PS	Spherical	505 ± 60	Structures consistent with PS-NPs observed attached to root surface as primarily individual particles with minimal aggregation across unwashed, washed, and sonicated (10 and 30 min) conditions; particles could not be fully removed by washing or sonication, indicating strong surface adhesion rather than loose contamination	[64]
FE-SEM	Phoebe bournei (stem)	Stem segments (0.5 cm) → frozen in liquid nitrogen → freeze-dried → gold-coated (60 s, ~1 nm) → imaged	PS	—	30 (29.99 ± 1.81)	NPs observed accumulated on the cell wall of xylem in stem tissue	[65]
TEM	Chrysanthemum coronarium (root, leaf)	Fresh tissue cut into small pieces → pre-fixed in 2.5% glutaraldehyde → washed (3×) with PBS → post-fixed in 1% osmium tetroxide → dehydrated (graded ethanol series, 30–100%) → embedded in Spurr’s resin (overnight) → ultrathin sectioning (microtome)	PS	—	—	Structures consistent with PS-MPs observed within root and leaf cells	[56]
TEM	Rice (roots)	2.5% glutaraldehyde fixation → embedding → cured at 70 °C → ultrathin sectioning → copper mesh	PS	Spherical	200	Suspected MPs particles observed within root cells	[50]
TEM	Lettuce (leaves and roots)	Fixation (glutaraldehyde) → post-fixation (osmium tetroxide) → dehydration (graded ethanol series) → resin embedding (Spurr’s resin) → ultrathin sectioning → staining (uranyl acetate, lead citrate)	PS	Spherical	93.6	Structures consistent with PS-NPs observed in leaf chloroplasts and as aggregates in root tissue, suggesting possible downward translocation	[42]
TEM	Rye (leaf)	Rinsed with PBS → fixed in 2.5% glutaraldehyde → post-fixed in 1% osmium tetroxide → rinsed with PBS → dehydrated (graded ethanol series) → embedded in epoxy resin → sectioned → stained with uranyl acetate → mounted on copper grids	PS	—	—	Structures consistent with NPs uptake into leaf cells revealed; treated cells showed enlarged, flattened chloroplasts, disrupted thylakoid arrangement, increased starch granules, and altered plastoglobuli number/size	[54]
CLSM	Cotton (roots)	Roots washed → embedded in 4% agarose → sectioned (50 µm, transverse and longitudinal) → mounted on glass slide with coverslip → soaked in PBS	PS (PS-COOH)	—	—	Fluorescent PS-COOH observed progressively in intercellular spaces of epidermis (5 h), epidermis and cortex (10 h), xylem vessels (16 h), and xylem, epidermis, and cortex together (24 h), indicating apoplastic uptake and vascular transport	[46]
CLSM	Tomato (leaves—mesophyll, protoplast cells)	Leaves rinsed with deionized water and sonicated to remove surface-adsorbed NPs → cut into 0.5 cm^2^ slices; protoplasts washed with sterile PBS → mounted on slide with coverslip	F-PS-SO_3_H/F-PS-NH_2_	—	—	F-PS-NH_2_ penetrated mesophyll cells more efficiently, distributed uniformly, and entered protoplasts as internalized clusters; F-PS-SO_3_H showed limited internalization (entering leaf cells only by 24 h) and aggregated around, rather than inside, protoplasts	[39]
CLSM	Phoebe bournei (root, shoot)	Tissue subsections (2 cm) embedded in 5% agarose → sectioned (15 µm, vibrating blade microtome) → imaged at 488/550 nm excitation/emission	PMMA (fluorescently labelled)	—	—	PMMA fluorescence accumulated in root endodermis and xylem, and in corresponding lignified/cuticle-enriched tissues of the shoot	[65]
CLSM	Garden cress (root, stem, leaf)	Rinsed with distilled water → sectioned → mounted on glass slide with distilled water and coverslip	PS	—	—	Fluorescent PS-NPs observed in root, stem, and leaf tissues; predominantly in root and leaf intercellular spaces and stem vascular tissue (stele); aggregation and heterogeneous distribution observed; 13–18% translocated to aerial parts relative to root accumulation	[30]
CLSM	Tomato (root)	Roots washed, air-dried → cut into ~0.2 mm thick slices → mounted on glass slide with glycerol → covered with coverslip → imaged	PVC	—	2.7–4.4	PVC-MPs visualized embedded in root cortex; high-intensity fluorescent particles present in treated roots, absent (beyond natural autofluorescence) in control roots	[57]
CLSM	Cucumber (roots)	Fresh roots cleaned with deionized water → mature root zone placed on glass slide with water → covered with cover glass	PS	—	—	Fluorescent PS-NPs visualized in cucumber roots across four tested particle sizes (100–700 nm); particles localized in epidermis and cortical intercellular spaces; no fluorescence observed in control	[66]
CLSM	Wheat (root tips)	Fresh tissue, no fixation → direct mounting on glass slide	PS	—	—	Fluorescent PS-NPs observed in root tips; leaf signal could not be distinguished from tissue autofluorescence	[58]

CLSM, confocal laser scanning microscopy; FE-SEM, field emission-scanning electron microscopy; MPs, microplastics; NPs, nanoparticles; NMPs, nano- and microplastics; PBS, phosphate-buffered saline; PS, polystyrene; PS-MPs, polystyrene-microplastics; PS-NPs, polystyrene-nanoparticles; PET, polyethylene terephthalate; PET–fSPIONs, polyethylene terephthalate–functionalized with superparamagnetic iron oxide nanoparticles; PVC, polyvinyl chloride; PLA, polylactic acid; SEM, scanning electron microscopy; TEM, transmission electron microscopy; PS-Eu, Europium-doped polystyrene; PMMA, poly(methyl methacrylate); PS-COOH, carboxyl-modified polystyrene; F-PS-SO_3_H, fluorescently labelled sulfonate-modified polystyrene; F-PS-NH_2_, fluorescently labelled amine-modified polystyrene; mg mL^−1^, milligrams per millilitre; nm, nanometre; μm, micrometre; cm^2^, square centimetre; °C, degree Celsius.

**Table 2:** Comparative analytical characteristics of microscopy and spectroscopic techniques used for detecting and characterizing nano- and microplastics (NMPs) in plant tissue, summarizing resolution, chemical identification capability, key strengths, and limitations.

Technique	Resolution Level	Chemical/ Polymer Identification	Quantitative Capability	Key Strength	Key Limitation
SEM	Few to tens of nanometres	No	Limited (particle counts/size distribution within imaged field only)	Reveals particle shape and surface attachment	Cannot confirm chemical or polymer identity on its own
FE-SEM	Few nanometres (finer than SEM)	No	Limited (calibration-based tissue concentration estimation demonstrated in specific studies)	Improved resolution of nanoscale particle–tissue surface interactions	Identifies particles by morphology alone; cannot confirm polymer identity
TEM	Sub-nanometre (finest of all techniques compared)	No	Limited (individual particle dimensions only; not bulk quantification)	Resolves internal ultrastructure and subcellular localization	Requires extensive sample preparation; very small field of view; cannot confirm chemical composition on its own
CLSM	Few hundred nanometres (coarser than electron microscopy techniques)	Confirms presence of a fluorescent label, not the polymer itself	Semi-quantitative (fluorescence intensity-based; particle-number estimation demonstrated in specific cases)	Enables 3D visualization of larger tissue volumes	Relies on fluorescent labelling
FTIR (point-based)	Point/bulk measurement	Yes	No (identification-focused, not quantification)	Confirms chemical/polymer identity at a single point	Localized measurement only; not spatially mapped across tissue
Raman spectroscopy	Point/micro-scale	Yes	No (identification-focused, not quantification)	Confirms chemical/polymer identity; complements FTIR	Localized measurement; not mapped across tissue
AFM-IR	Nanoscale (hybrid technique)	Yes	No (identification-focused, not quantification)	Combines chemical identification with nanoscale surface topography	Point-based; not tissue-wide mapping
SR-FTIR	Spatially resolved, tissue scale	Yes	No	Extends chemical confirmation across intact tissue	Cannot determine plastic quantity
VNIR/NIR-HSI	Wide-field, tissue/plant scale	Indirect biochemical/ physiological signal only	Yes, for plant stress indices—not polymer quantity	Detects plant stress responses associated with NMPs exposure	Does not confirm polymer identity directly
MIR-HSI	Spatially resolved, tissue scale	Yes	No	Provides spatial location and chemical identity from a single acquisition	Not yet demonstrated in plant tissue; cannot determine plastic quantity
ICP-MS	No spatial resolution (bulk/digested tissue analysis)	No (elemental detection; requires elemental labelling of particles)	Yes, quantitative (concentration measured directly in digested tissue)	Direct, quantitative tracking of elementally labelled particle uptake and translocation	Cannot describe particle location, shape, or morphology within tissue
Py-GC/MS	No spatial resolution (bulk/pyrolyzed tissue analysis)	Yes (polymer identified via characteristic thermal degradation fragments)	Yes, quantitative (mass-based concentration with recovery/detection limits reported)	Confirms both polymer identity and quantity from a single destructive analysis	Requires extensive sample pretreatment; destroys sample; no spatial or morphological information

SEM, scanning electron microscopy; FE-SEM, field emission-scanning electron microscopy; TEM, transmission electron microscopy; CLSM, confocal laser scanning microscopy; FTIR, Fourier-transform infrared; AFM-IR, atomic force microscopy–infrared spectroscopy; SR-FTIR, synchrotron radiation-Fourier-transform infrared; VNIR/NIR-HSI, visible/near-infrared-hyperspectral imaging; MIR-HSI, mid-infrared-hyperspectral imaging; ICP-MS, inductively coupled plasma–mass spectrometry; Py-GC/MS, pyrolysis-gas chromatography/mass spectrometry.

**Table 3:** Overview of the analytical stages involved in detecting and characterizing nano- and microplastics (NMPs) in plant tissue, showing the research question addressed at each stage and the corresponding techniques discussed in this review.

Research Stage	Research Question	Main Techniques
Morphological imaging	Where are the particles and what do they look like?	SEM, FE-SEM, TEM, CLSM
Chemical identification	Are the particles really plastics?	FTIR, Raman spectroscopy, AFM-IR
Chemical imaging	Where are specific polymers located within tissues?	SR-FTIR, MIR-HSI
Quantitative analysis	How much plastic is present and what is its composition?	Py-GC/MS, ICP-MS

SEM, scanning electron microscopy; FE-SEM, field emission-scanning electron microscopy; TEM, transmission electron microscopy; CLSM, confocal laser scanning microscopy; FTIR, Fourier-transform infrared; AFM-IR, atomic force microscopy–infrared spectroscopy; SR-FTIR, synchrotron radiation-Fourier-transform infrared; MIR-HSI, mid-infrared-hyperspectral imaging; ICP-MS, inductively coupled plasma–mass spectrometry; Py-GC/MS, pyrolysis-gas chromatography/mass spectrometry.

## Data Availability

No new data were created or analyzed in this study. Data sharing is not applicable.
